# Biosynthetic route towards saxitoxin and shunt pathway

**DOI:** 10.1038/srep20340

**Published:** 2016-02-04

**Authors:** Shigeki Tsuchiya, Yuko Cho, Keiichi Konoki, Kazuo Nagasawa, Yasukatsu Oshima, Mari Yotsu-Yamashita

**Affiliations:** 1 Graduate School of Agricultural Science, Tohoku University, 1-1 Tsutsumidori-Amamiya, Aoba-ku, Sendai 981-8555, Japan; 2 Faculty of Technology, Tokyo University of Agriculture and Technology, 2-24-16 Naka-cho, Koganei-shi, Tokyo 184-8588, Japan; 3 Graduate School of Life Sciences, Tohoku University, 2-1-1 Katahira, Aoba-ku, Sendai 980-8577, Japan

## Abstract

Saxitoxin, the most potent voltage-gated sodium channel blocker, is one of the paralytic shellfish toxins (PSTs) produced by cyanobacteria and dinoflagellates. Recently, putative biosynthetic genes of PSTs were reported in these microorganisms. We previously synthesized genetically predicted biosynthetic intermediates, Int-A’ and Int-C’2, and also Cyclic-C’ which was not predicted based on gene, and identified them all in the toxin-producing cyanobacterium *Anabaena circinalis* (TA04) and the dinoflagellate *Alexandrium tamarense* (Axat-2). This study examined the incorporation of ^15^N-labeled intermediates into PSTs (C1 and C2) in *A. circinalis* (TA04). Conversions from Int-A’ to Int-C’2, from Int-C’2 to Cyclic-C’, and from Int-A’ and Int-C’2 to C1 and C2 were indicated using high resolution-LC/MS. However, Cyclic-C’ was not converted to C1 and C2 and was detected primarily in the extracellular medium. These results suggest that Int-A’ and Int-C’2 are genuine precursors of PSTs, but Int-C’2 converts partially to Cyclic-C’ which is a shunt product excreted to outside the cells. This paper provides the first direct demonstration of the biosynthetic route towards saxitoxin and a shunt pathway.

Saxitoxin (STX, **1**) (Fig. 1) and its analogues^1^,^2^, which are known as the paralytic shellfish toxins (PSTs), are strong and selective voltage-gated sodium channel (Na_v_Ch) blockers^3^,^4^. In addition to this specific biological activity, a unique tricyclic structure with two guanidines for PSTs, consisting of more heteroatoms than carbon atoms, has attracted biologists and chemists^5^,^6^,^7^,^8^,^9^,^10^. PSTs are produced by several species of freshwater cyanobacteria^11^,^12^,^13^,^14^,^15^,^16^,^17^,^18^,^19^,^20^,^21^ and marine dinoflagellates^22^,^23^,^24^,^25^,^26^, which cause serious damage to human and animal health and to seafood production worldwide. The biosynthesis of STX was first studied by Shimizu *et al*.^27^,^28^,^29^,^30^, who conducted feeding experiments with ^13^C- and/or ^15^N-labeled amino acids and acetic acid using the marine dinoflagellate *Gonyaulax tamarensis* (at present, *Alexandrium tamarense*), and the freshwater cyanobacterium *Aphanizomenon flos-aquae*. Shimizu *et al*.^27^,^28^,^29^,^30^ indicated that acetic acid, arginine and *S*-adenosyl methionine are the components to build up PSTs molecules, and proposed a biosynthetic pathway towards STX, including the Claisen condensation of arginine and acetic acid. Then, Kellmann *et al*.^31^ discovered putative STX biosynthetic gene clusters (*sxt*) in the cyanobacterium *Cylindrospermopsis raciborskii* T3 and proposed a different route from Shimizu’s route based on the genetic sequences (Fig. 1). They also suggested the presence of Int-A’ (**3**), Int-C’ (structurally different from Int-C’2 (**4**)) and Int-E’ (**6**) (Fig. 1) in this cyanobacterium by MS/MS spectra^31^. Eventually, a core set of 14 genes (*sxtA-sxtI, sxtP-sxtR, sxtS, and sxtU*) was commonly found in the PSTs-producing cyanobacteria. In the marine dinoflagellates, the presence of the similar genes possibly corresponding to PSTs biosynthesis were first reported by Stüken *et al*.^32^ in two PSTs-producing dinoflagellate strains (*Alexandrium fundyense* CCMP1719 and *A. minutum* CCMP113).

In our previous study^33^,^34^, we synthesized the genetically predicted biosynthetic intermediates proposed by Kellmann *et al*.^31^, Int-A’ (**3**) and Int-C’2 (**4**), as well as the related compound, Cyclic-C’ (**7**) (Fig. 1), and identified them in the PST-producing cyanobacterium *Anabaena circinalis* (TA04)^15^ and the dinoflagellate *Alexandrium tamarense* (Axat-2)^35^,^36^,^37^ using high resolution (HR)-LC/MS. These compounds were not detected in the non-PST-producing cyanobacterium *Anabaena circinalis* (NIES-1645)^38^ and dinoflagellate *Alexandrium tamarense* (UAT-014-009)^35^,^36^,^37^, supporting the suggestion that these compounds are implicated in the STX biosynthesis. However, it has not been determined that Int-A’ (**3**), Int-C’2 (**4**), and Cyclic-C’ (**7**) are the genuine precursors or shunt products of PSTs. In the PSTs biosynthesis, shunt pathways have not yet been reported, although they are potentially important to reduce the PSTs production. In this study, we conducted experiments to incorporate [^15^N_2_]-labeled Int-A’ (**3′**), Int-C’2 (**4′**) and Cyclic-C’ (**7′**) into the major STX analogues (PSTs), C1 (**8**) and C2 (**9**)^39^ (Fig. 2a), using *A. circinalis* (TA04). Furthermore, we also examined the intracellular and extracellular concentrations of these compounds to support the results of incorporation experiments.

## Results and Discussion

[2,6-^15^N_2_]Arginine (**2′**), [2,6-^15^N_2_]Int-A’ (**3′**), [2,7-^15^N_2_]Int-C’2 (**4′**) and [3,9-^15^N_2_]Cyclic-C’ (**7′**) were synthesized from [2,5-^15^N_2_]L-ornithine by following the synthetic route we reported previously (Fig. 3)^33^,^34^.

**Figure 1:**
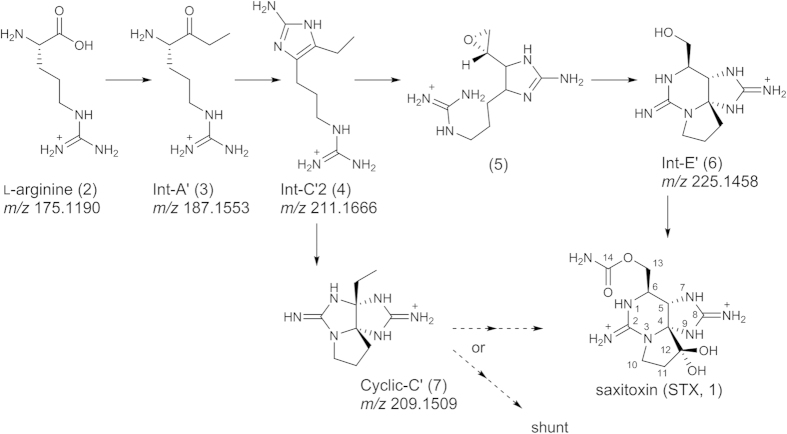
The biosynthetic route towards saxitoxin (STX, 1) and intermediates (2, 3, 4, 5, 6) proposed by Kellmann *et al*.^31^ with 7, which was found by us^34^. Molecules **3**, **4** and **7** were identified in the cyanobacterium *A. circinalis* (TA04) and the dinoflagellate *A. tamarense* (Axat-2), and the structure of **4** was drawn as we reported^33^,^34^.

**Figure 2:**
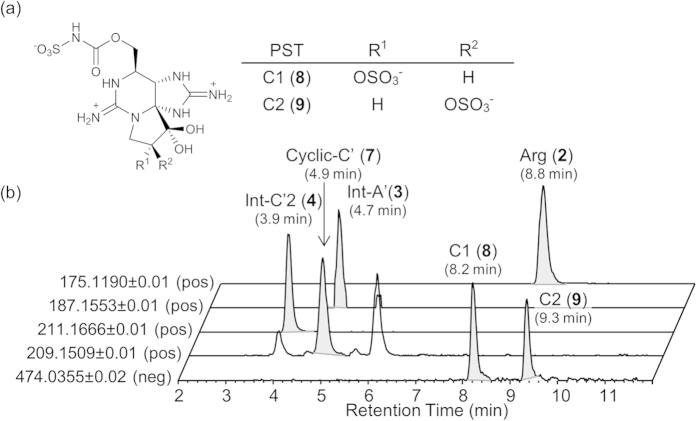
HR-LC/MS analysis of Arg (2), intermediates (3, 4, 7), and PSTs (C1 (8)/C2 (9)). (**a**) Chemical structures of C1 (**8**) and C2 (**9**)^39^. (**b**) Typical HR-LC/MS extracted ion chromatograms (EICs) for Arg (**2**), Int-A’ (**3**), Int-C’2 (**4**), Cyclic-C’ (**7**) (positive mode), C1 (**8**) and C2 (**9**) (negative mode) in the extracts from the cells of *A. circinalis* (TA04).

[2,6-^15^N_2_]Int-A’ (**3′**), [2,7-^15^N_2_]Int-C’2 (**4′**) and [3,9-^15^N_2_]Cyclic-C’ (**7′**) were independently administered into the culture medium of *A. circinalis* (TA04) at 5 μM, and cultivation was continued for 7 days. At concentrations higher than 5 μM, the cytotoxic effects of these compounds were observed in *A. circinalis* (TA04). In the cells cultured without labeled compounds (control), arginine (**2**), Int-A’ (**3**), Int-C’2 (**4**), and Cyclic-C’ (**7**) were detected at *m/z* 175.1190 (8.8 min), 187.1553 (4.7 min), 211.1666 (3.9 min), and 209.1509 (4.9 min), respectively, as the [M + H]^ + ^ions in the positive mode using HR-LC/MS (Fig. 2b). When [^15^N_2_]Int-A’ (**3′**) was administered, the ions at *m/z* 213 ([^15^N_2_]Int-C’2, 3.9 min) (**4′**) and at *m/z* 211 ([^15^N_2_]Cyclic-C’, 4.9 min) (**7′**) were prominently shown (Fig. 4b). This result indicated that the ^15^N_-_label from [^15^N_2_]Int-A’ (**3′**) was incorporated into [^15^N_2_]Int-C’2 (**4′**) and [^15^N_2_]Cyclic-C’ (**7′**) by bioconversion with the ratios of 88% and 97%, respectively, based on the peak areas. Similarly, when [^15^N_2_]Int-C’2 (**4′**) was administered, the intensity of the ion at *m/z* 211 ([^15^N_2_]Cyclic-C’, 4.9 min) (**7′**) clearly increased (Fig. 4c), suggesting that [^15^N_2_]Int-C’2 (**4′**) was converted to [^15^N_2_]Cyclic-C’ (**7′**) with the ^15^N-labeling ratio of 97%. As expected, Int-A’ (**3**) and Int-C’2 (**4**) were not ^15^N-labeled from [^15^N_2_]Cyclic-C’ (**7′**) (Fig. 4d). The conversions from [^15^N_2_]Int-A’ (**3′**) to [^15^N_2_]Int-C’2 (**4′**) and from [^15^N_2_]Int-C’2 (**4′**) to [^15^N_2_]Cyclic-C’ (**7′**) were further confirmed by the MS/MS fragmentation patterns using HR-LC-MS/MS (see Fig. S1). The possibility of decomposition of the ^15^N-labeled compounds administered in the medium was excluded because the arginine (**2**), located upstream of Int-A’ (**3**), Int-C’2 (**4**), and Cyclic-C’ (**7**) (Fig. 1), was not ^15^N-labeled, and random labeling of the compounds was not suggested by the MS results. Therefore, these results supported conversion from Int-A’ (**3**) to Int-C’2 (**4**), from Int-C’2 (**4**) to Cyclic-C’ (**7**), and from Int-A’ (**3**) to Cyclic-C’(**7**) via Int-C’2 (**4**).

Next, incorporation of ^15^N-labels from [^15^N_2_]Int-C’2 (**4′**) and [^15^N_2_]Cyclic-C’ (**7′**) into PSTs (C1 (**8**) and C2 (**9**)) was examined. C1 (**8**) and C2 (**9**)^39^ (Fig. 2a) are the major PSTs in *A. circinalis* (TA04)^15^. These compounds are easily epimerized with each other at C-11 due to the keto-enol tautomerism of the ketone at C-12^40^. C2 (**9**) was reported to have been biosynthesized and then partially converted to C1 (**8**) spontaneously^40^. C1 (**8**) and C2 (**9**) are predicted to be derived from STX (**1**) in this cyanobacterium because such derivation was demonstrated in the dinoflagellate *Gymnodinium catenatum*^41^,^42^. Using LC/MS, C1 (**8**) and C2 (**9**) were detected at 8.2 min and 9.3 min, respectively, at *m/z* 474.0355 (monoisotopic ion) as the [M-H]^−^ ion in the negative mode (Fig. 2b) due to the presence of two SO_3_^−^ groups. As the preliminary test, the cells of *A. circinalis* (TA04) were cultured in the medium and independently administered [^15^N_2_]Int-A’ (**3′**), [^15^N_2_]Int-C’2 (**4′**), or [^15^N_2_]Cyclic-C’ (**7′**) at 5 μM for 16 days. Then, the ^15^N-labeled ratios of C1 (**8**) and C2 (**9**) were estimated based on the unlabeled (**8**, **9**) (*m/z* 474) and ^15^N-labeled (**8′**, **9′**) (*m/z* 476) peak areas on the spectra of HR-LC/MS (Fig. S2). In these estimations, the influence of the theoretical natural abundance ratio of *m/z* 476 to *m/z* 474 (12.4%) was excluded. The ratios in the samples administered [^15^N_2_]Int-A’ (**3′**), [^15^N_2_]Int-C’2 (**4′**), and [^15^N_2_]Cyclic-C’ (**7′**) were 4%, 6%, 0%, respectively, whereas the value of the control (without administration of any labeled compounds) was 0%. These values were too low to judge whether the labeled compounds were incorporated or not. Therefore, [^15^N_2_]Int-C’2 (**4′**) or [^15^N_2_]Cyclic-C’ (**7′**) were repetitively added to the medium of *A. circinalis* (TA04). A half-volume of the one-month culture of TA04 was harvested, and the same volume of the fresh medium containing [^15^N_2_]Int-C’2 (**4′**) or [^15^N_2_]Cyclic-C’ (**7′**) (final concentration at 5 μM) was administered into the remaining culture approximately every 10 days for 31 days (0-, 10-, 20- or 21-day). In each harvested sample, the ratios of the relative intensities of the isotopic peak of *m/z* 476 to *m/z* 474 corresponding to C1 (**8**) and C2 (**9**) were examined and compared with each other (C2 (**9**): Fig. 5, C1 (**8**): Fig. S3). As expected, the ratios of [^15^N_2_]-labeled C1 (**8′**) and C2 (**9′**) increased at every addition of [^15^N_2_]Int-C’2 (**4′**) for 31 days (Fig. 5a,c and Fig. S3a,c). The [^15^N_2_]-labeled ratios of C2 (**9**) at 0, 10, 20 and 31 days were 0%, 11%, 13% and 17%, respectively (Fig. 5c), whereas the ratios of C1 (**8**) at day 0, 10, 20 and 30 were 0%, 5%, 13% and 17% (Fig. S3c), respectively. Identification of [^15^N_2_]-labeled C2 (**9′**) was further supported by HR-LC-MS/MS pattern that was similar to unlabeled C2 (**9**) (Fig. S4).

In contrast, when [^15^N_2_]Cyclic-C’ (**7**′) was administered to the medium repetitively, the labeled ratio of C2 (**9**) did not increase at all until day 31 (Fig. 5b,c), suggesting that Cyclic-C’ (**7**) was not converted to C2 (**9**). In addition, the incorporation ratio of ^15^N-labels from [^15^N_2_]Int-C’2 (**4′**) into [^15^N_2_]C2 (**9′**) was approximately 17% at day 31 (Fig. 5a,c), even if [^15^N_2_]Int-C’2 (**4′**) was administered repetitively. According to these results, we assumed that when surplus [^15^N_2_]Int-C’2 (**4′**) (5 μM in this experiment) was administered, [^15^N_2_]Int-C’2 (**4′**) was, for the most part, converted into [^15^N_2_]Cyclic-C’ (**7′**) (Fig. 6, route-b), which is not converted to STX analogues ([^15^N_2_]C1 (**8′**) and [^15^N_2_]C2 (**9′**)). However, a part of [^15^N_2_]Int-C’2 (**4′**) was converted to STX analogues (PSTs), [^15^N_2_]C1 (**8′**) and [^15^N_2_]C2 (**9′**) (Fig. 6, route-a). Thus, Cyclic-C’ (**7**) can be considered a shunt product. These explanations are summarized in Fig. 6.

To confirm this assumption, intracellular and extracellular concentrations of Int-A’ (**3**), Int-C’2 (**4**) and Cyclic-C’ (**7**) were analyzed every two days until day 10 for *A. circinalis* (TA04) cultured under normal conditions. As shown in Fig. 7, the concentrations of Int-A’ (**3**) and Int-C’2 (**4**) were higher in the cells than in the medium from day 2 to day 10. In contrast, the extracellular concentration of Cyclic-C’ (**7**) was higher than its intracellular concentration from day 4 to day 10 (Fig. 7c). In addition, the concentration of total Cyclic-C’ (**7**) was much higher than the concentration of total Int-C’2 (**4**) in all experimental periods. These results supported the above assumption that excessively administered Int-C’2 (**4**) (at 5 μM in this experiment) was converted to Cyclic-C’ (**7**) and released from the cells into the culture medium, while a small part of the administered Int-C’2 (**4**) was converted to PSTs (C1 (**8**) and C2 (**9**)) (Fig. 6). Thus, Cyclic-C’ (**7**) might be a shunt metabolite to reduce PSTs production. In PSTs non-producing dinoflagellates^32^,^43^,^44^ and cyanobacteria^45^, partial deletion of the *sxtA* gene or a complete lack of the sxt gene cluster have been reported as explanations for failure to produce toxin. However, to the best of our knowledge, conversion from Int-C’2 to Cyclic-C’ (**7**) is the first shunt pathway in PSTs producing microorganisms.

As we reported previously, the concentrations of C1 (**8**) and C2 (**9**) were much higher than the concentrations of the intermediates, **3**, **4**, and **7** under normal cultural conditions^33^,^34^. Indeed, the intracellular concentrations of C1 (**8**) and C2 (**9**) at day 10 in this experiment were 1.03 and 0.99 μM, respectively, whereas the intracellular concentrations of Int-A’ (**3**), Int-C’2 (**4**), and Cyclic-C’ (**7**) were only of the 0.1–10 nM order, as shown in Fig. 7. The result of almost complete ^15^N-labeling of the intermediates (**4** and **7,** Fig. 4) might be derived from the lower concentrations of **3**, **4**, and **7** than their administered concentrations (5 μM) in the starting culture. Lower ^15^N-labeled ratios of **8** and **9** (final 17%) were probably caused by higher concentrations of unlabeled C1 (**8**) and C2 (**9**) provided from the pre-culture in the starting culture.

**Figure 3:**
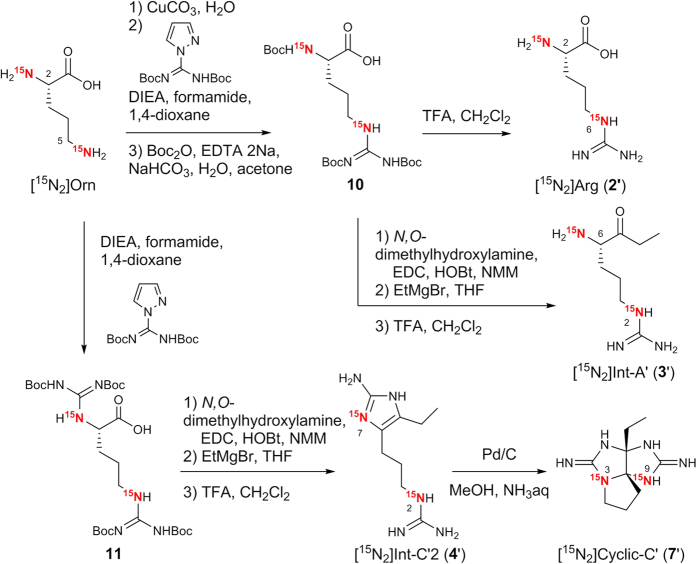
Synthesis of ^15^N-labeled arginine (2′) and biosynthetic intermediates (3′, 4′, 7′)^33^,^34^.

**Figure 4:**
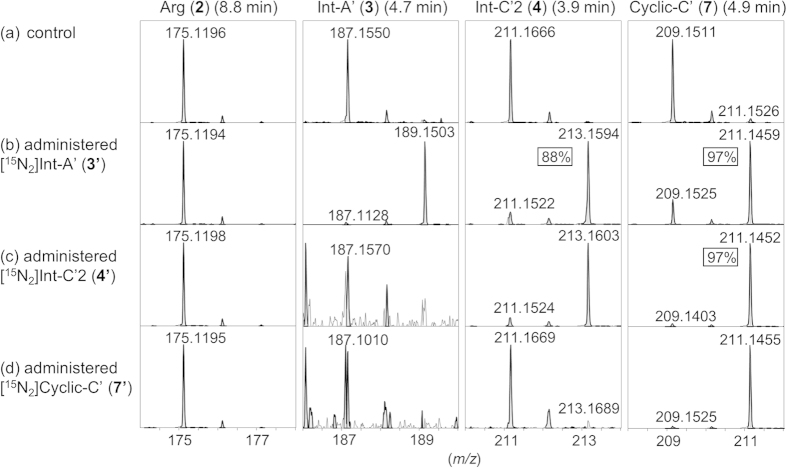
Incorporation of ^15^N-labeled intermediates (3′, 4′, 7′) into the other intermediates in the cells of *A. circinalis* (TA04). Isotopic patterns of Arg (**2**), Int-A’ (**3**) Int-C’2 (**4**) and Cyclic-C’ (**7**) in the cell extracts of the 7-day culture of control (not administered) (**a**) and ^15^N-labeled intermediates (**3′**, **4′**, **7′**) independently administered in cultures at 5.0 μM (**b–d**), analyzed using LC/MS. [M + H]^ + ^ions for the authentic [^15^N_2_]Int-C’2 (**4′**) and [^15^N_2_]Cyclic-C’ (**7′**) were detected at *m/z* 213.1606 and 211.1450, respectively. The ^15^N-labels incorporated ratios were shown in the spectra.

**Figure 5:**
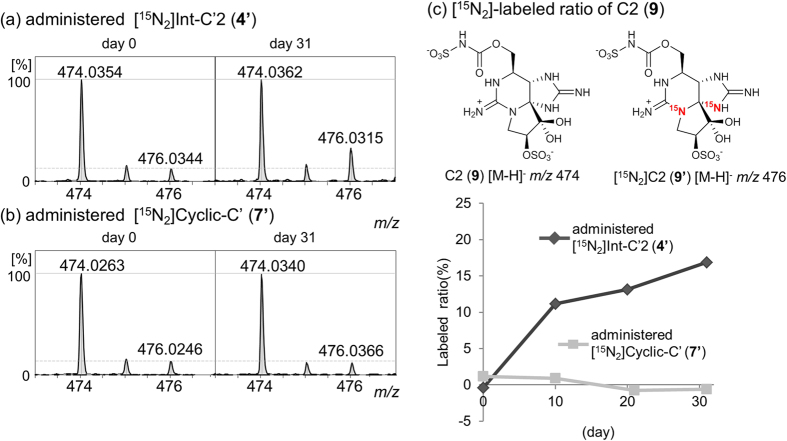
Incorporation of ^15^N-labels into C2 (9) in the cells. [^15^N_2_]Int-C’2 (**4′**) or [^15^N_2_]Cyclic-C’ (**7′**) were repetitively administered to the medium at 5.0 μM approximately every 10 days for 31 days (0-, 10-, 20- or 21-day). (**a,b**) Isotope patterns of C2 (**9**) at day 0 and day 31. The abundance of *m/z* 474 is shown as 100%. The theoretical natural abundance of *m/z* 476 (12.4%) is shown as a dashed line. (**c**) [^15^N_2_]-labeled ratio of C2 (**9**).

**Figure 6:**
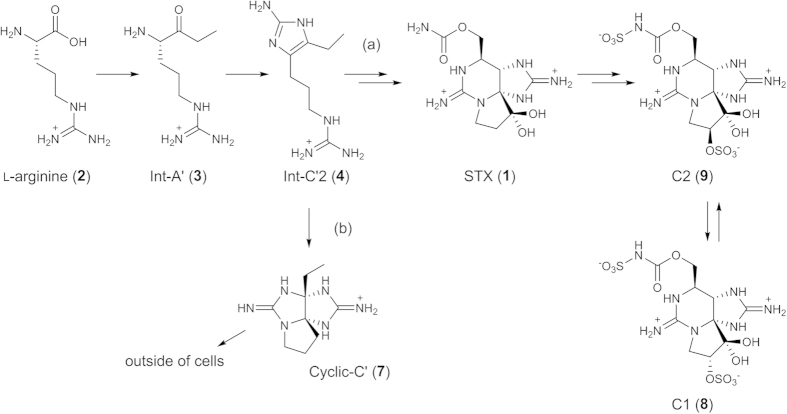
The biosynthetic route towards PSTs (**a**) and shunt pathway (**b**) in *A. circinalis* (TA04) suggested in this study.

**Figure 7:**
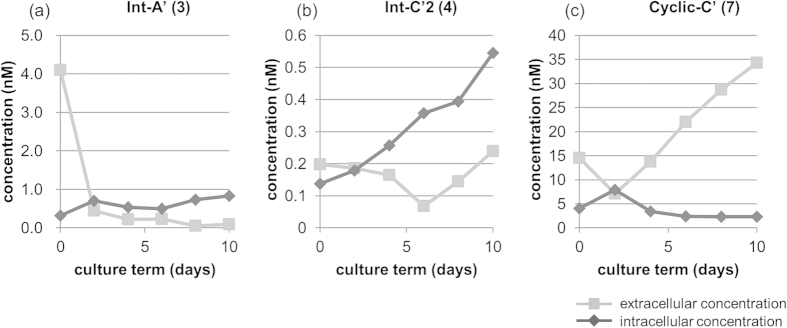
Comparison between intracellular and extracellular concentrations. Intracellular concentrations are calculated as cells that are suspended in the medium.

We also administered 5–200 μM [^15^N_2_]arginine (**2′**) (Fig. 3) into the culture medium of *A. circinalis* (TA04) and cultured this cyanobacterium for 3–7 days under the same condition as the feeding experiments using **3′**, **4′**, and **7′** (see, Methods). However, Int-A’ (**3**), Int-C’2 (**4**), Cyclic-C’ (**7**), C1 (**8**), and C2 (**9**) were not labeled. In addition, [^15^N_2_]arginine (**2′**) was not detected in the cells, although unlabeled these compounds were all similarly detected as those shown in Fig. 4a. Shimizu *et al*. reported that [2-^13^C-2-^15^N]arginine was incorporated into saxitoxin derivatives using the cyanobacterium *Aphanizomenon flos-aquae*, although the experimental detail was not described^30^. We assume that this discrepancy was caused by the difference of the experimental condition.

The results in this study demonstrated the genetically predicted biosynthetic route to STX (**1**) proposed by Kellmann *et al*.^31^ and are also consistent with the results of feeding experiments reported by Shimizu *et al*.^27^,^28^,^29^,^30^. Several biologically important microbial shunt pathways have been reported^46^,^47^. Shunt pathways in saxitoxin biosynthesis are potentially important to reduce the PSTs productions. Although investigation of the Cyclic-C’ (**7**) shunt pathway in the dinoflagellate is still underway, we previously reported the presence of Cyclic-C’ (**7**) in the PSTs producing *Alexandrium tamarense* (Axat-2). Furthermore, the studies of the latter stage of the biosynthetic pathway towards PSTs are in progress using other synthetic intermediates.

In conclusion, we demonstrated that genetically predicted biosynthetic intermediates, Int-A’ (**3**) and Int-C’2 (**4**), are true precursors of PSTs (C1 (**8**) and C2 (**9**)), while Cyclic-C’ (**7**), which is partially converted from Int-C’2 (**4**), is a shunt product to be released from the cells to the outside to reduce PSTs production. This report presents the first evidence to demonstrate this route and the first finding of the shunt pathway for PSTs production. Biosynthetic steps for production of PSTs in the later stage have not been fully elucidated. This finding will accelerate efforts to clarify the full process of PSTs biosynthesis.

## Methods

### General

The isotopically labeled ornithine, [2,5-^15^N_2_]L-ornithine HCl, was purchased from Cambridge Isotope Laboratories, Inc. (Tewksbury, MA, USA). The dry solvents for organic synthesis were purchased from Wako Pure Chemical Industries, Ltd. (Osaka, Japan). The other reagents were purchased from Sigma-Aldrich Co. (St. Louis, MO, USA), Wako Pure Chemical Industries, Ltd., Tokyo Chemical Industry Co., Ltd. (Tokyo, Japan), and Nacalai Tesque, Inc. (Kyoto, Japan). LC/MS-grade acetonitrile (Wako Pure Chemical Industries, Ltd.) was used for LC-Q-TOF MS. Distilled and purified water (MilliQ) by Simplicity UV (Merck Millipore Corporation, Billerica, MA, USA) was used for all the experiments. NMR spectra were recorded with Agilent 600 MHz NMR spectrometer (Agilent Technologies, Inc., Santa Clara, CA, USA) with D_2_O or CD_3_OD as the solvent and internal standard. Spectra were referenced to residual solvent signals with resonances at δ_H_ = 4.79 ppm (D_2_O), δ_H/C_ = 3.30/49.8 ppm (CD_3_OD) and at δ_N_ = 0 ppm (NH_3_). LC/MS was performed with micrOTOF-Q II (ESI, Q-TOF) (Bruker Daltonics Inc, Billerica, MA, USA) and API2000 (AB SCIEX, Foster City, CA, USA). High resolution (HR) MS was measured with a micrOTOF-Q II (ESI). The basic culture conditions for *A. circinalis* (TA04) are same as we reported previously^33^.

### Synthesis of ^15^N-labeled arginine (2′) and intermediates (3′, 4′, 7′)

**[2,6-**^**15**^**N**_**2**_**]L-arginine (2′): 2**′ was obtained as TFA salt. ^1^H NMR (600 MHz, D_2_O): δ = 4.02 (H-2, t, *J*_H,H_ = 6 Hz, 1 H), 3.17 (H-5, t, *J*_H,H_ = 7.2 Hz, 2 H), 1.92 (H-3, m, 2 H), 1.65 ppm (H-4, m, 2 H); ^13^C NMR (151 MHz, D_2_O): δ = 171.6 (C-1), 156.5 (C-7), 52.3 (C-2), 40.1 (C-5), 26.7 (C-3), 23.6 ppm (C-4); ^15^N NMR (60.8 MHz, D_2_O) 82.6 (N-6), 37.3 ppm (N-2); HRMS (ESI^ + ^) (*m/z*): [M + H]^ + ^calcd. for C_6_H_15_N_2_^15^N_2_O_2_, 177.1130; found, 177.1131.

**[2,6-**^**15**^**N**_**2**_**]Int-A’ (3′): 3**′ was obtained as 2 TFA salt. ^1^H NMR (600 MHz, CD_3_OD): δ = 4.15 (H-6, dd, *J*_H,H_ = 7.3, 4.1 Hz, 1 H), 3.19 (H-3, t, *J*_H,H_ = 6.6 Hz, 2 H), 2.65 (H-8, m, 1 H), 2.54 (H-8, m, 1 H), 2.02 (H-5, m, 1 H), 1.82 (H-5, m, 1 H), 1.67 (H-4, m, 1 H), 1.55 (H-4, m, 1 H), 1.05 ppm (H-9, t, *J*_H,H_ = 7.2 Hz, 3 H); ^13^C NMR (151 MHz, CD_3_OD): δ = 208.0 (C-7), 159.6 (C-1), 60.1 (C-6), 42.5 (C-3), 34.0 (C-8), 28.7 (C-5), 26.1 (C-4), 8.3 ppm (C-9); ^15^N NMR (60.8 MHz, CD_3_OD) 81.5 (N-2), 31.8 ppm (N-6); HRMS (ESI^ + ^) (*m/z*): [M + H]^ + ^calcd. for C_8_H_19_N_2_^15^N_2_O, 189.1494; found, 189.1512.

**[2,7-**^**15**^**N**_**2**_**]Int-C’2 (4′): 4**′ was obtained as 2 TFA salt. ^1^H NMR (600 MHz, CD_3_OD): δ = 3.18 (H-3, t, *J*_H,H_ = 6.8 Hz, 2 H), 2.53 (H-5, td, *J*_H,H_ = 7.9, 2.7 Hz, 2 H), 2.47 (H-11, q, *J*_H,H_ = 7.6 Hz, 2 H), 1.82 (H-4, m, 2 H), 1.17 ppm (H-12, t, *J*_H,H_ = 7.5 Hz, 3 H); ^13^C NMR (151 MHz, CD_3_OD): δ = 159.5 (C-1), 148.8 (C-8), 126.1 (C-10), 121.8 (C-6), 42.2 (C-3), 30.0 (C-4), 22.1 (C-5), 16.5 (C-11), 15.0 ppm (C-12), ^15^N NMR (60.8 MHz, CD_3_OD) 133.0 (N-7), 81.5 (N-2); HRMS (ESI^ + ^) (*m/z*): [M + H]^ + ^calcd. for C_9_H_19_N_4_^15^N_2_, 213.1606; found, 213.1616.

**[3,9-**^**15**^**N**_**2**_**]Cyclic-C’ (7′): 7′** was obtained as 2 formic acid salt after HPLC purification using reversed phase column. ^1^H NMR (600 MHz, CD_3_OD): δ = 3.53 (H-10, m, 1 H), 3.42 (H-10, m, 1 H), 2.39 (H-11, m, 1 H), 2.36 (H-11, m, 1 H), 2.28 (H-12, m, 1 H), 2.18 (H-12, m, 1 H), 2.12 (H-6, m, 1 H), 2.03 (H-6, m, 1 H), 1.05 (H-13, t, *J*_H,H_ = 7.4 Hz, 3 H); ^13^C NMR (151 MHz, CD_3_OD): δ = 161.3 (C-2), 159.7 (C-8), 95.5 (C-4), 85.6 (C-5), 48.1 (C-10), 32.5 (C-12), 29.8 (C-6), 27.3 (C-11), 8.6 ppm (C-13), ^15^N NMR (60.8 MHz, CD_3_OD) 114.9 (N-3), 104.4 (N-9); HRMS (ESI^ + ^) (*m/z*): [M + H]^ + ^calcd. for C_9_H_17_N_4_^15^N_2_, 211.1450; found, 211.1447.

### Repetitive administration of ^15^N-labeled intermediates and preparation of the samples for HR-LC/MS (Q-TOF)

To a 40 mL preliminary culture of *A. circinalis* (TA04), 40 mL of the fresh CB’ medium^48^ and 40 μL of 10 mM [^15^N_2_]-Int-C’2 (**4′**) or 10 mM [^15^N_2_]Cyclic-C’ (**7′**) in MeOH were independently added and cultured for 31 days. During cultivation, half of the culture (40 mL) was harvested, and the fresh CB’ medium (40 mL) and 40 μL of 10 mM solutions of **4′** or **7′** were added to the remaining culture approximately every 10 days. The cells were suspended with 2.0 mL of 0.5 M AcOH and sonicated three times for 30 s on ice, and the resulting solutions were centrifuged at 20,000 *g* for 5 min at 4 °C. The supernatants were filtered through a Cosmospin filter H (0.45 μm, Nacalai Tesque, Kyoto, Japan). The filtrates were used for two different analyses, that is, for PSTs and intermediates. A part (100 μL) of the filtrate was adjusted to pH 5 using 2 M NH_3_ aq, and loaded on an activated charcoal column (500 μL vol.). After the column was washed with H_2_O, PSTs were eluted with AcOH/EtOH/H_2_O (5:50:45, v/v/v). The solvent was removed using N_2_ gas and lyophilized, and the resulting residue was resuspended with 100 μL of 0.5 M AcOH as an analytical sample for PSTs. Another 100 μL of the filtrate through the Cosmospin filter H was adjusted to pH 7–8 using 2 M NH_3_ aq, and loaded on a Cosmosil 140C18-OPN column (100 mg). After the column was washed with H_2_O, intermediates were eluted with AcOH/MeOH/H_2_O (5:50:45, v/v/v). The solvent was removed using N_2_ gas and lyophilized, and the resulting residue was resuspended with 100 μL of 0.5 M AcOH for the analysis of intermediates.

### HR-LC/MS analysis

The mass analysis was performed based on our previous reports^33^,^34^. The liquid chromatography system used for analysis was a Shimadzu Nexera UHPLC System (Shimadzu, Kyoto, Japan). The autosampler (SIL-30AC, Shimadzu) was maintained at 5 °C. Liquid chromatography was performed on a 2.1 i.d. × 150 mm (130 Å, 1.7 μm) ACQUITY UPLC BEH Amide column (Waters, Milford, MA, USA). Mobile phase A was 200 mM HCOONH_4_ buffer containing 200 mM HCOOH/H_2_O (5:95, v/v, pH 3.9), and mobile phase B was 200 mM HCOONH_4_ buffer containing 200 mM HCOOH/H_2_O /CH_3_CN (5:1.5:95, v/v/v). A gradient elution program was applied as follows: 0–3 min 85% B, 3–11 min 85–70% B, 11–13 min 70% B, 13–20 min 85% B. The flow rate was 0.3 mL/min. The oven temperature was 40 °C. The liquid chromatography system was connected to a Q-TOF mass spectrometer, MicrOTOF-QII (Bruker Daltonics, Billerica, MA, USA), equipped with an ESI source. The mass spectrometer conditions were as follows: positive (for Arg (**2**) and intermediates (**3**, **4**, **7**)) or negative (for C1 (**8**) and C2 (9)) ionization mode; dry gas: nitrogen at 10 L/min; dry temperature: 180 °C; nebulizer: 2.2 bar; capillary: 4500 V (for Arg and intermediates) or −4500 V (for C1 (**8**) and C2 (**9**)). The peaks for Arg (**2**), Int-A’ (**3**), Int-C’2 (**4**), Cyclic-C’ (**7**), C1 (**8**) and C2 (**9**) were detected at 8.8 min, 4.7 min, 3.9 min,4.9 min, 8.2 min and 9.3 min, respectively.

HR-LC-MS/MS for the labeled intermediates (**4′**, **7′**) in AutoMSMS mode and C8 (**9′**) in MRM mode was performed in setting [M + H]^ + ^or [M-SO_3_ + H]^ + ^as the precursor ions. The precursor ions were *m/z* 213.16 for [2,7-^15^N_2_]Int-C’2 (**4′**) and 211.15 for [3,9-^15^N_2_]Cyclic-C’ (**7′**) width 4 Da, and [M-SO_3_ + H]^ + ^*m/z* 398.09 and *m/z* 396.09 for the ^15^N-labeled C2 (**9′**) and C2 (**9**), respectively, width 3 Da. The sweeping collision energy was 40–120 eV for **4′** and **7′**, and 15–30 eV for **9** and **9′**. The mass spectrometer conditions were as follows: positive ionization mode; dry gas: nitrogen at 7.0 L/min; dry temperature: 180 °C; nebulizer: 1.6 bar; capillary: 4500 V.

### The comparison of intracellular and extracellular concentrations of Int-A’ (3), IntC’2 (4), and Cyclic-C’ (7)

The preliminary culture (150 mL) was mixed with 150 mL of fresh CB’ medium to start the culture. A 30 mL culture of *A. circinalis* (TA04) was harvested every other day until day 10. The cells and the medium were separated by filtration using the glass fiber filter (GA100, 1.0 μm, Advantec, Tokyo, Japan). Analytical samples of the cells were prepared according to our previous report^33^,^34^. Analytical samples of the medium were prepared from the filtrates of GA100. A part of the filtrate (8 mL) was loaded on a Cosmosil 140C18-OPN (100 mg) packed in a glass pipette, which had been conditioned with MeOH and water, then washed with 2 mL of water. Int-A’ (**3**), Int-C’2 (**4**), and Cyclic-C’ (**7**) were eluted with 2 mL of MeOH/H_2_O/AcOH (50:45:5, v/v/v). The eluate was concentrated *in vacuo* and redissolved with 400 μL of 0.5 M AcOH. Compounds **3**, **4**, and **7** were quantified using the column-switching LC-MS/MS (MRM) method using API200 mass spectrometer^33^,^34^. The recoveries of **3**, **4**, and **7** were 48%, 37%, and 73%, respectively, for this procedure, determined by the spiked medium. The final concentrations of the intermediates were calculated by considering the above recovery rates.

## Additional Information

**How to cite this article**: Tsuchiya, S. *et al*. Biosynthetic route towards saxitoxin and shunt pathway. *Sci. Rep*. **6**, 20340; doi: 10.1038/srep20340 (2016).

## Supplementary Material

Supplementary Information
