# Supplementary material for: Biosynthetic route towards saxitoxin and shunt pathway
Source: Sci Rep. 2016 Feb 4;6:20340. doi: 10.1038/srep20340 (PMC4740887; doi:10.1038/srep20340)

Supplementary information

Biosynthetic route towards saxitoxin and shunt pathway

Shigeki Tsuchiya[1], Yuko Cho[1], Keiichi Konoki[1], Kazuo Nagasawa[2], Yasukatsu Oshima[3] and Mari Yotsu-Yamashita[1]*

[1]Graduate School of Agricultural Science, Tohoku University, 1-1 Tsutsumidori-Amamiya, Aoba-ku, Sendai 981-8555, Japan

[2]Faculty of Technology, Tokyo University of Agriculture and Technology, 2-24-16 Naka-cho, Koganei-shi, Tokyo 184-8588, Japan

[3]Prof. emeritus, Graduate School of Life Sciences, Tohoku University, 2-1-1 Katahira, Aoba-ku, Sendai 980-8577, Japan

Table of Contents

1.	Administration of ^{15}N-labeled intermediates into the culture medium of *A. circinalis*, and harvest	S2
2.	Recovery test of intermediates from the resin, Cosmosil 140C18-OPN	S2
3.	HR-LC-MS/MS spectra of [2,7-$^{15}N_2$]Int-C'2 (**4'**) and [3,9-$^{15}N_2$]Cyclic-C' (**7'**)	S3
4.	Isotopic patterns of C2 (**9**) in the cell extracts of the 16-day culture	S4
5.	Incorporation of ^{15}N-labels into C1 (**8**) in the cells	S5
6.	HR-LC-MS/MS spectra of C2 (**9**) and [$^{15}N_2$]C2 (**9'**) in MRM mode	S6
7.	NMR spectra of [2,6-$^{15}N_2$]Arg (**2'**)	S7-8
8.	NMR spectra of [2,6-$^{15}N_2$]Int-A' (**3'**)	S9-10
9.	NMR spectra of [2,7-$^{15}N_2$]Int-C'2 (**4'**)	S11-12
10.	NMR spectra of [3,9-$^{15}N_2$]Cyclic-C' (**7'**)	S13-14

Administration of ^{15}N-labeled intermediates into the culture medium of *A. circinalis*, and harvest.
To the preliminary culture of *A. circinalis* (TA04) in CB' medium (25 mL) were added 25 mL of fresh CB' medium and 25 µL of 10 mM [2,6-$^{15}N_2$]Int-A' (**3'**), [2,7-$^{15}N_2$]Int-C'2 (**4'**) or [3,9-$^{15}N_2$]Cyclic-C' (**7'**) in MeOH solution (independently). The cells cultured with aeration for 7 or 16 days were harvested by filtration using the glass fiber filter (GA100, 1.0 µm, Advantec, Tokyo, Japan), and lyophilized. Then, the preparations of the samples for HR-LC/MS (Q-TOF) were completed using same method.

Recovery test of intermediates from the resin, Cosmosil 140C18-OPN
A column filed with 100 mg of Cosmosil 140C18-OPN (Nacalai Tesque Inc. Kyoto, Japan) in a glass pipette was used for analysis of the medium except cells. The resin was conditioned with 2 mL of MeOH and 10 mL of water. A medium of *A. circinalis* (CB' medium, 8 mL) spiked authentic Int-A' (**3**), Int-C'2 (**4**), or Cyclic-C' (**7**) (0.2 µmol, respectively) was loaded, followed by a wash of 2 mL of water. Compounds **3**, **4**, and **7** were eluted with 2 mL of EtOH/water/AcOH (50:45:5, v/v/v), and the eluate was concentrated in vacuo. The test samples were redissolved with 400 µL of 0.5 M AcOH. Quantitation was performed using a column-switching HPLC-MS/MS method, previously reported[33,34]. The recoveries of **3**, **4**, and **7** were 48%, 37%, and 73%, respectively.

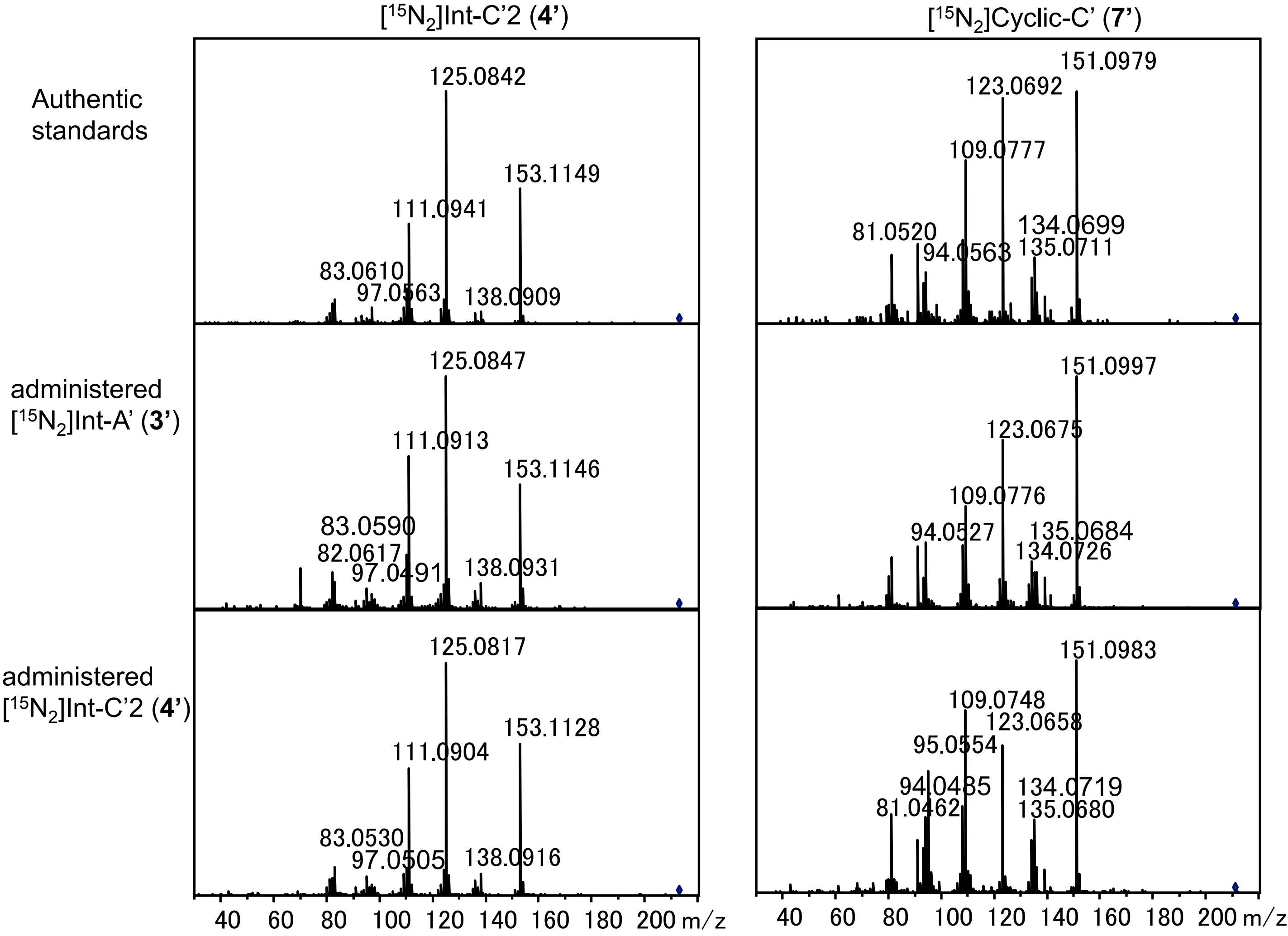

Figure S1. HR-LC-MS/MS spectra of [2,7-$^{15}N_2$]Int-C'2 (**4'**, 3.9 min) and [3,9-$^{15}N_2$]Cyclic-C' (**7'**, 4.9 min). The precursor ions were $[M+H]^+$ *m/z* 213.15 ([2,7-$^{15}N_2$]Int-C'2 (**4'**)) and 211.15 ([3,9-$^{15}N_2$] Cyclic-C' (**7'**)) width 4 Da. The sweeping collision energy was 40–120 eV.

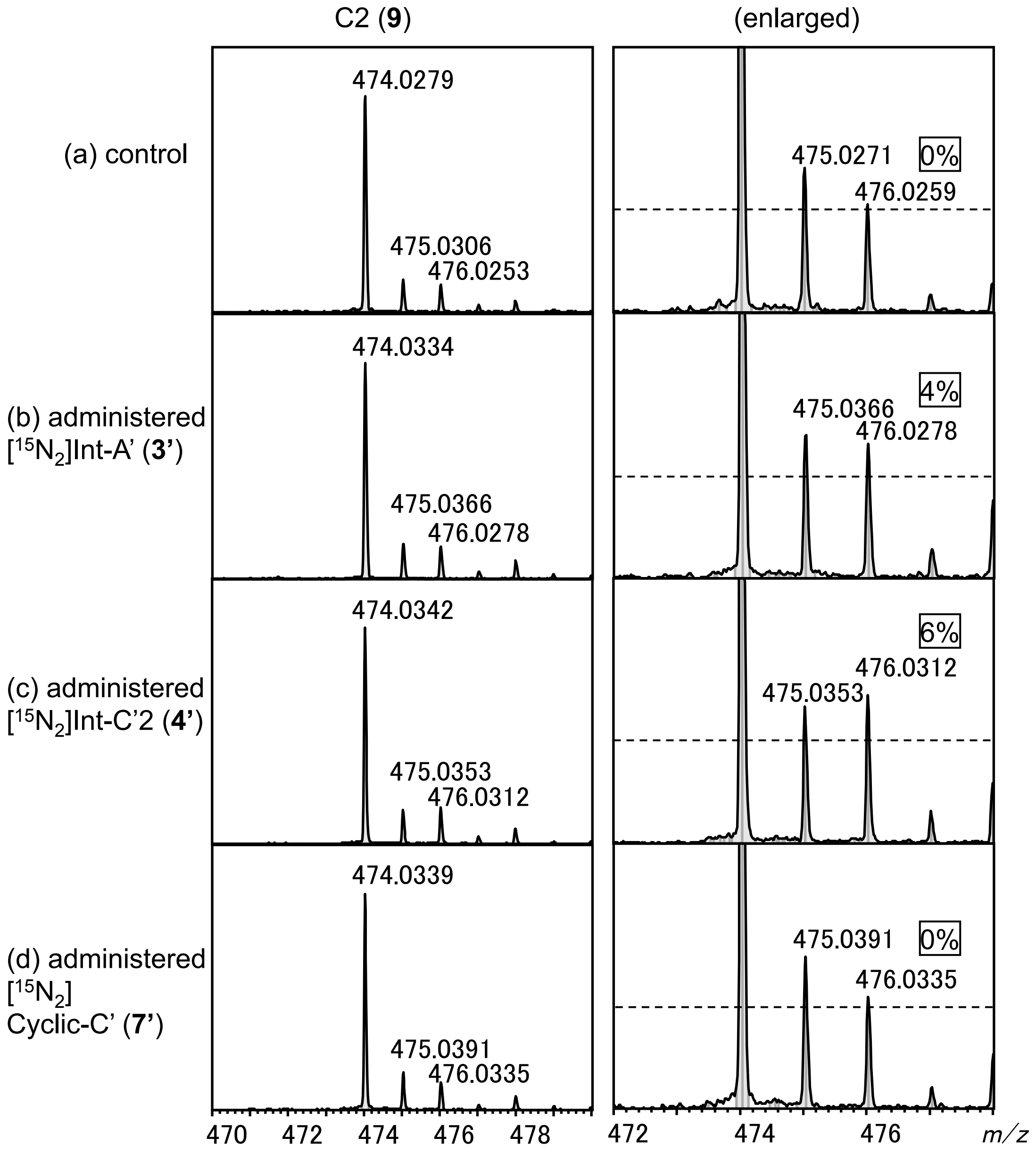

Figure S2. Isotopic patterns of C2 (**9**) in the cell extracts of the 16-day culture. (a) control (not administered), (b, c, d) ^{15}N-labeled compounds (**3'**, **4'**, **7'**) independently administered in culture at 5.0 μM, and the enlarged spectra with ^{15}N-labeled ratios. Dashed line: theoretical natural abundance of *m/z* 476 (12.4%) to *m/z* 474.

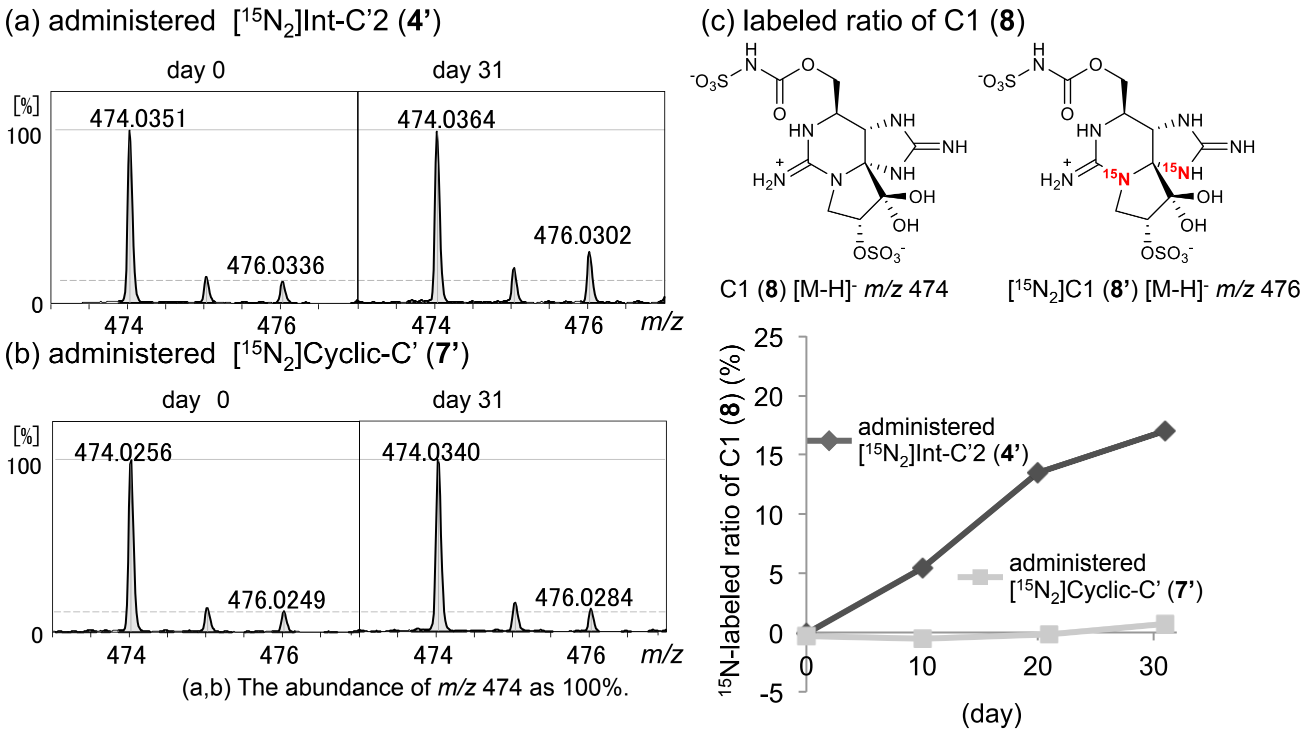

Figure S3. Incorporation of ^{15}N-labels into C1 (8) in the cells.
[$^{15}N_2$]Int-C'2 (**4'**) or [$^{15}N_2$]Cyclic-C' (**7'**) were repetitively administered to the medium at 5.0 μM approximately every 10 days for 31 days (0-, 10, 20- or 21-day). (a, b) Isotope patterns of C1 (**8**) at day 0 and day 31. The abundance of *m/z* 474 is shown as 100%. The theoretical natural abundance of *m/z* 476 (12.4%) to *m/z* 474 is shown as a dashed line. (c) [$^{15}N_2$]-labeled ratio of C1 (**8**).

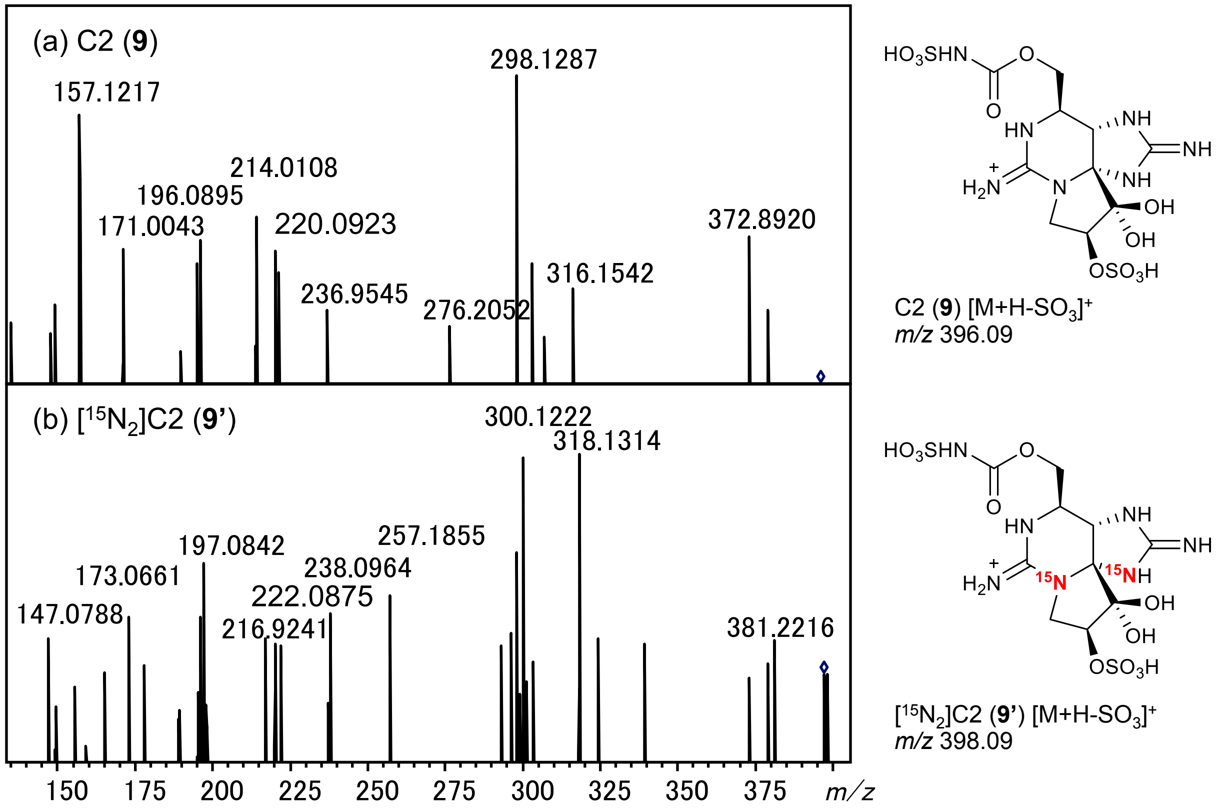

Figure S4. HR-LC-MS/MS spectra of C2 (**9**) and [$^{15}N_2$]C2 (**9'**) in MRM mode.
(a) C2 (**9**) at 0 day, (b) [$^{15}N_2$]C2 (**9'**) (9.1min) at 31 day after repeated administration of [$^{15}N_2$]Int-C'2 (**4'**). The precursor ions were $[M+H]^+$ *m/z* 396.09 (C2 (**9**)) (a) and 398.09 ([$^{15}N_2$]-C2 (**9'**)) (b) width 3 Da. The sweeping collision energy was 15–30 eV. The fragment ions at *m/z* 316.1542, 298.1287 of **9** and *m/z* 318.1314, 300.1222 of **9'** are assignable to their $[M\text{-}2SO_3\text{+}H]^+$ and $[M\text{-}2SO_3\text{-}H_2O\text{+}H]^+$, respectively.

[2,6-$^{15}N_2$]Arg (**2’**)
^{1}H NMR spectrum (600 MHz, D_2O)

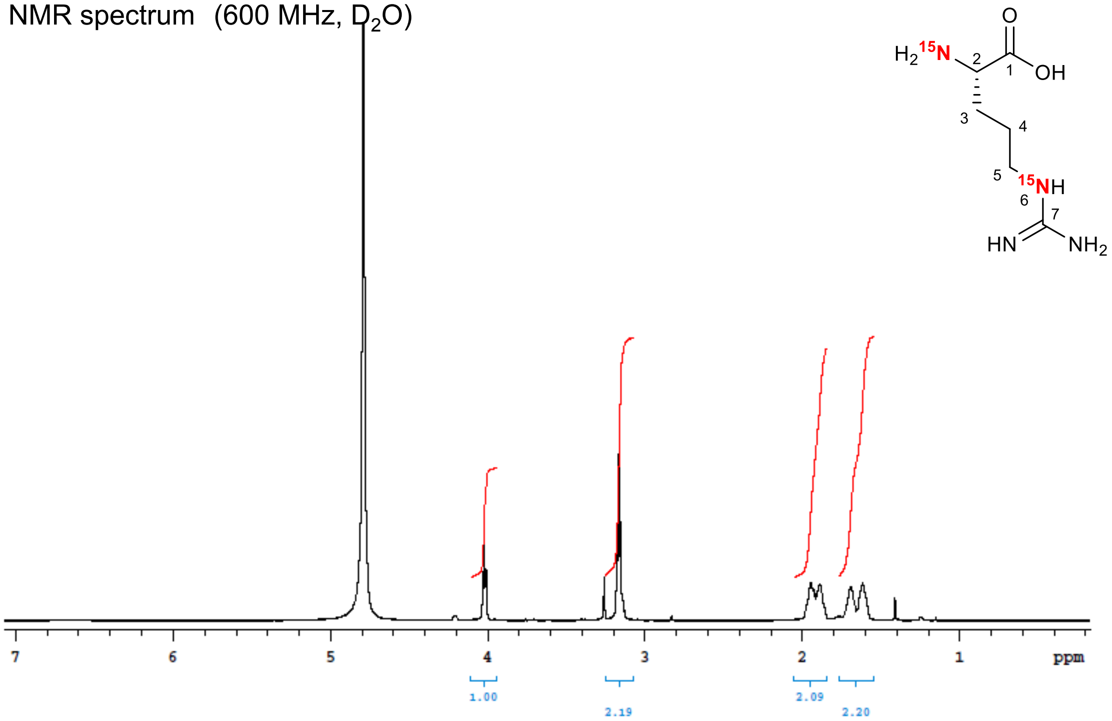

[2,6-$^{15}N_2$]Arg (**2’**)
^{13}C NMR spectrum (150 MHz, D_2O)

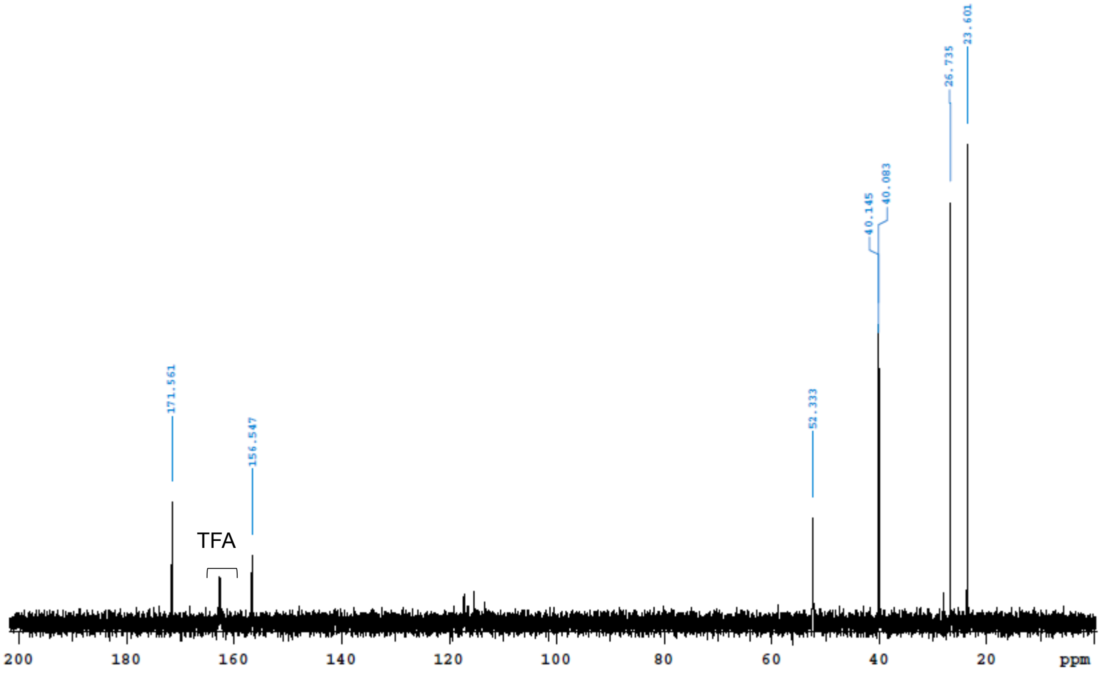

[2,6-$^{15}N_2$]Int-A' (**3'**)
^{15}N-^{1}H HMBC spectrum (CD_3OD)

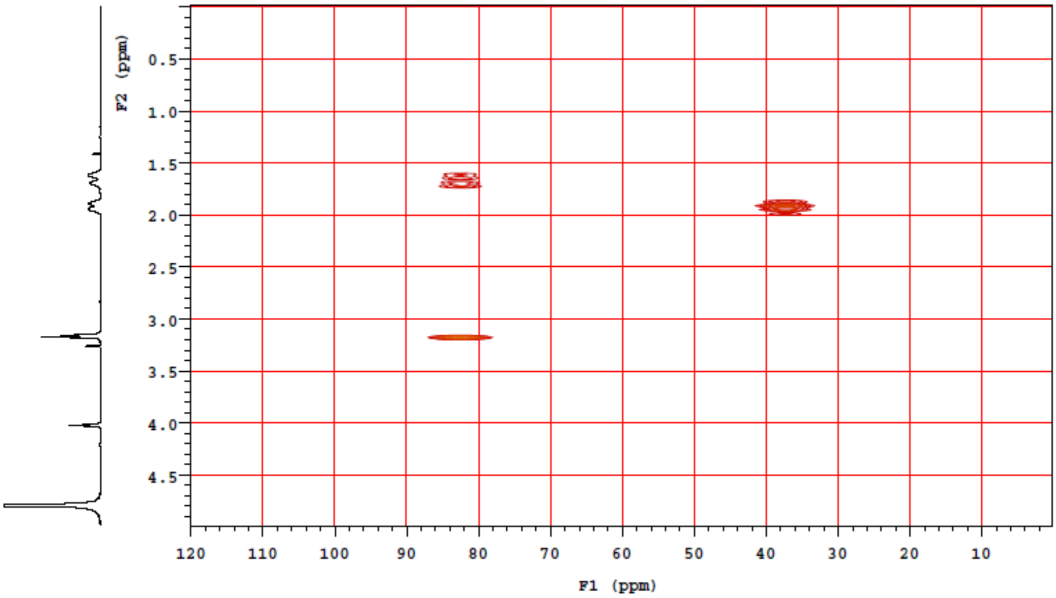

[2,6-$^{15}N_2$]Int-A' (**3'**)
^{1}H NMR spectrum (600 MHz, CD_3OD)

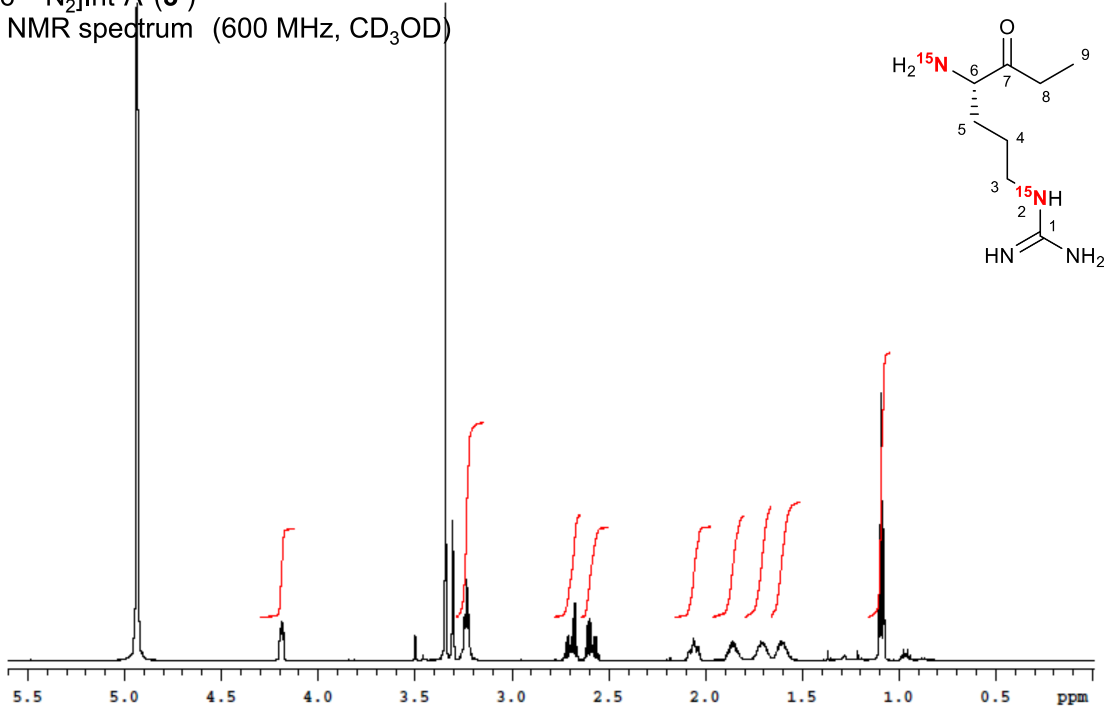

[2,6-$^{15}N_2$]Int-A' (**3'**)
^{13}C NMR spectrum (150 MHz, CD_3OD)

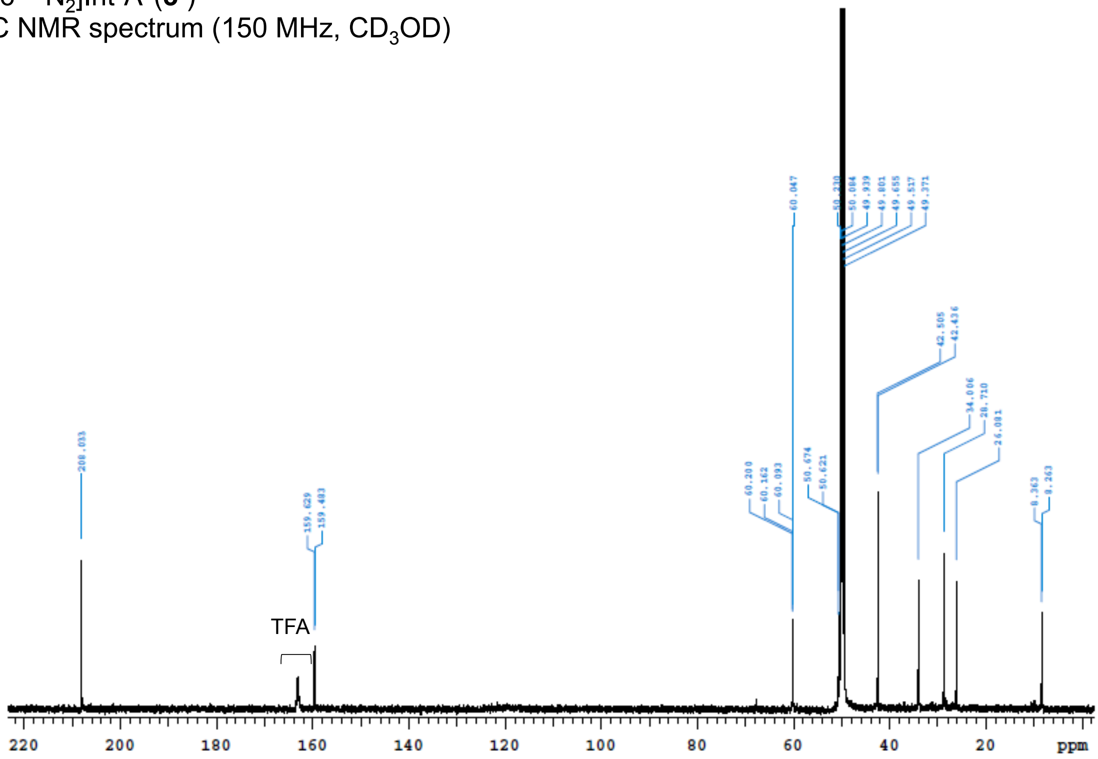

[2,6-$^{15}N_2$]Int-A' (**3'**)
^{15}N-^{1}H HMBC spectrum (CD_3OD)

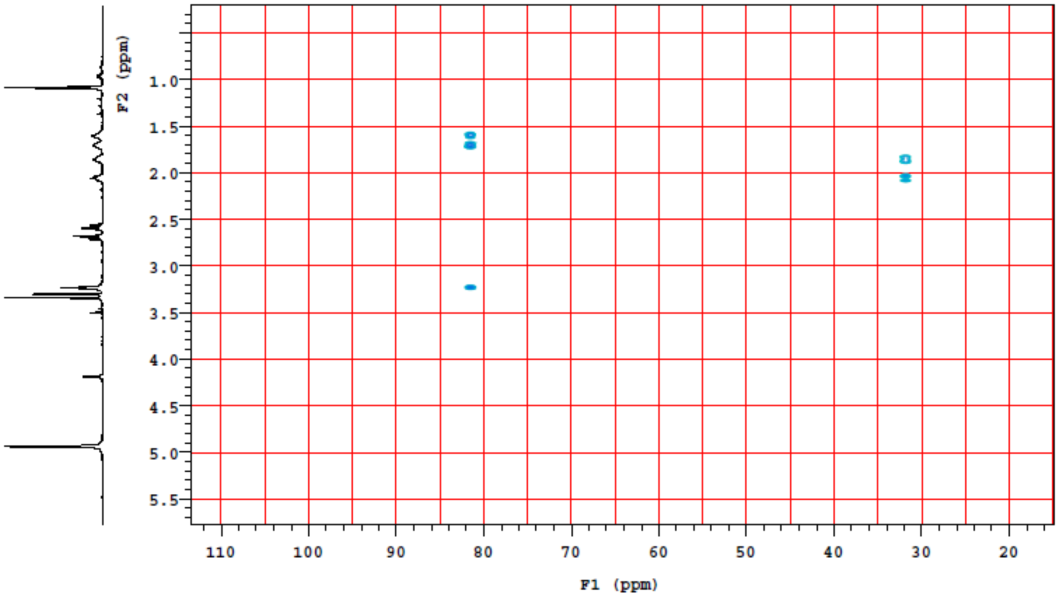

[2,7-$^{15}N_2$]Int-C'2 (**4'**)
^{1}H NMR spectrum (600 MHz, CD_3OD)

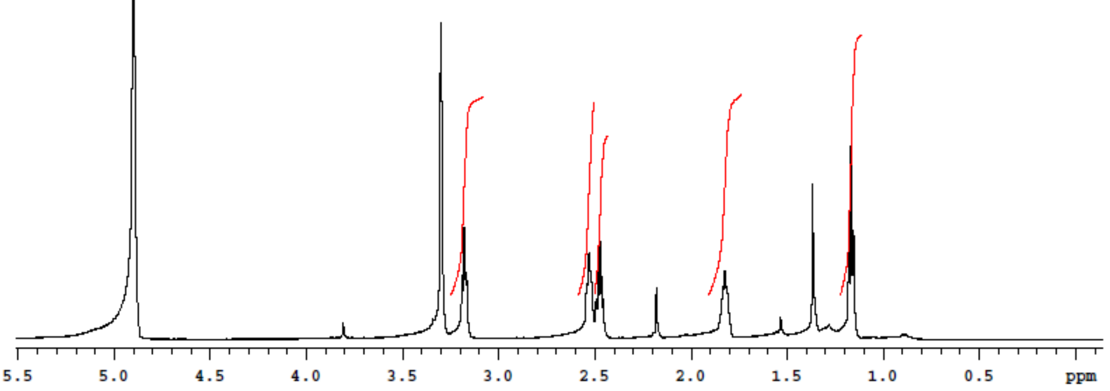

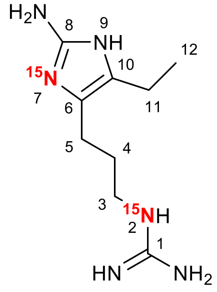

[2,7-$^{15}N_2$]Int-C'2 (**4'**)
^{1}H NMR spectrum (600 MHz, CD_3OD)

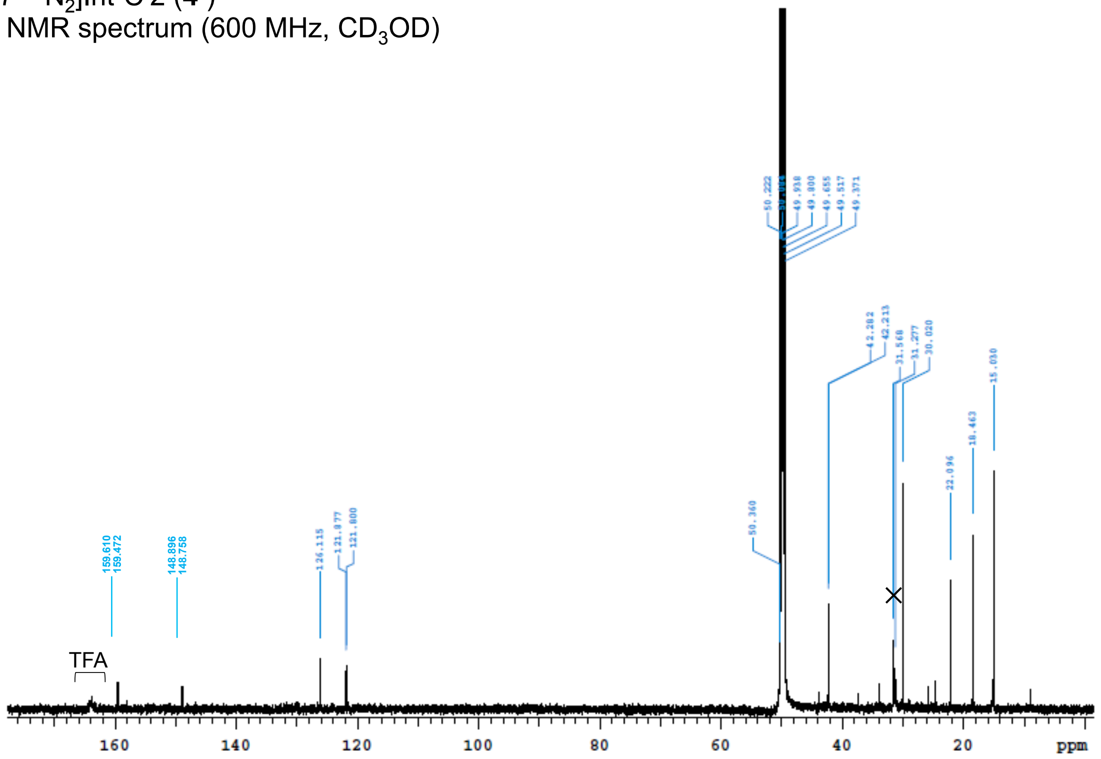

[2,7-$^{15}N_2$]Int-C'2 (**4'**)
^{15}N-^{1}H HMBC spectrum (CD_3OD)

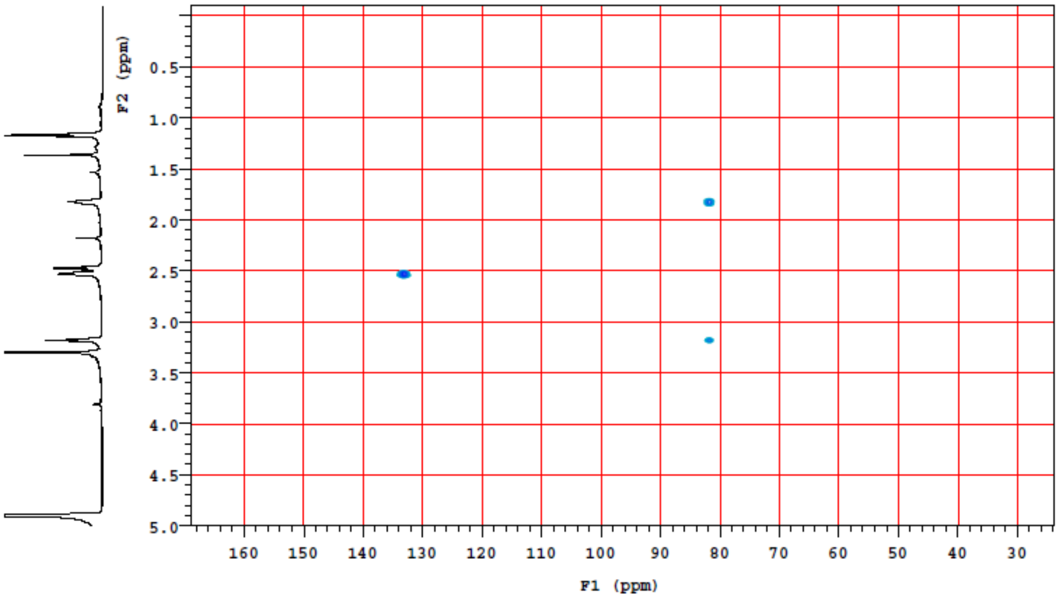

[3,9-$^{15}N_2$]Cyclic-C' (**7'**)
^1H NMR spectrum (600 MHz, CD_3OD)

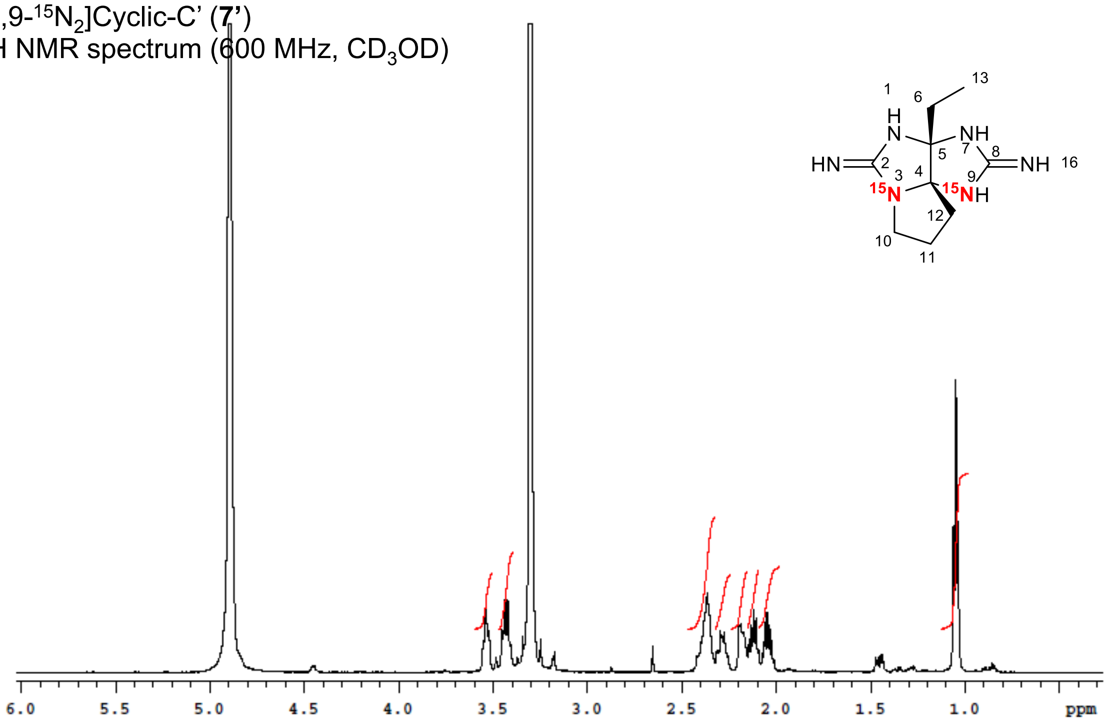

[3,9-$^{15}N_2$]Cyclic-C' (**7'**)
^{13}C NMR spectrum (150 MHz, CD_3OD)

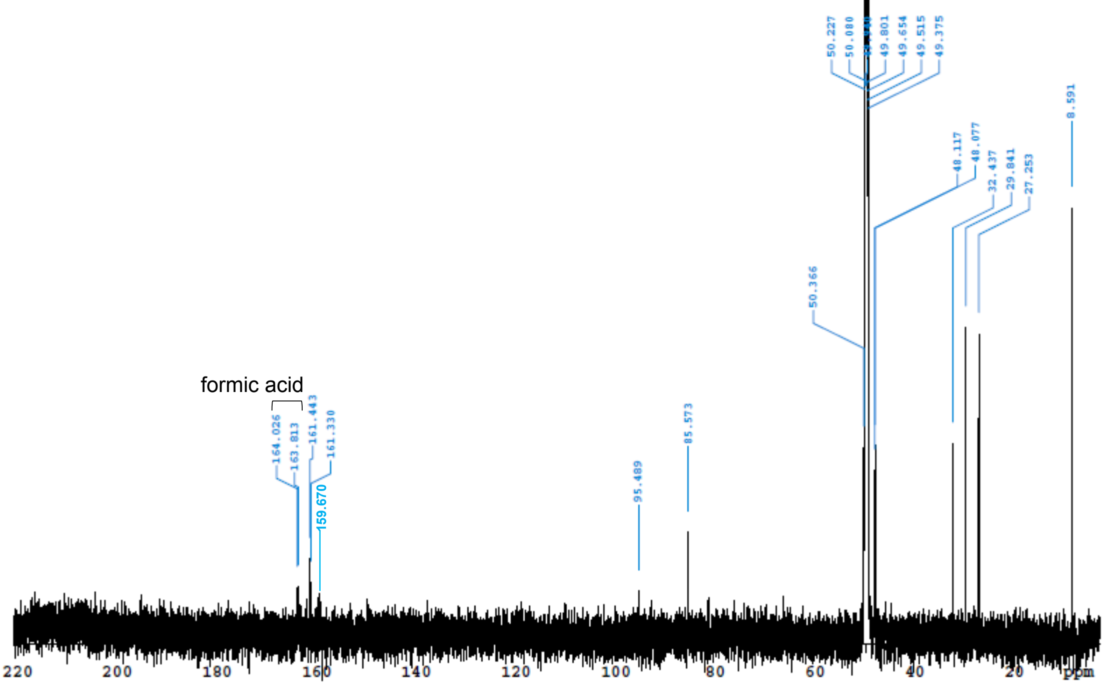

[3,9-$^{15}N_2$]Cyclic-C' (**7'**)
^{15}N-^{1}H HMBC spectrum (CD_3OD)

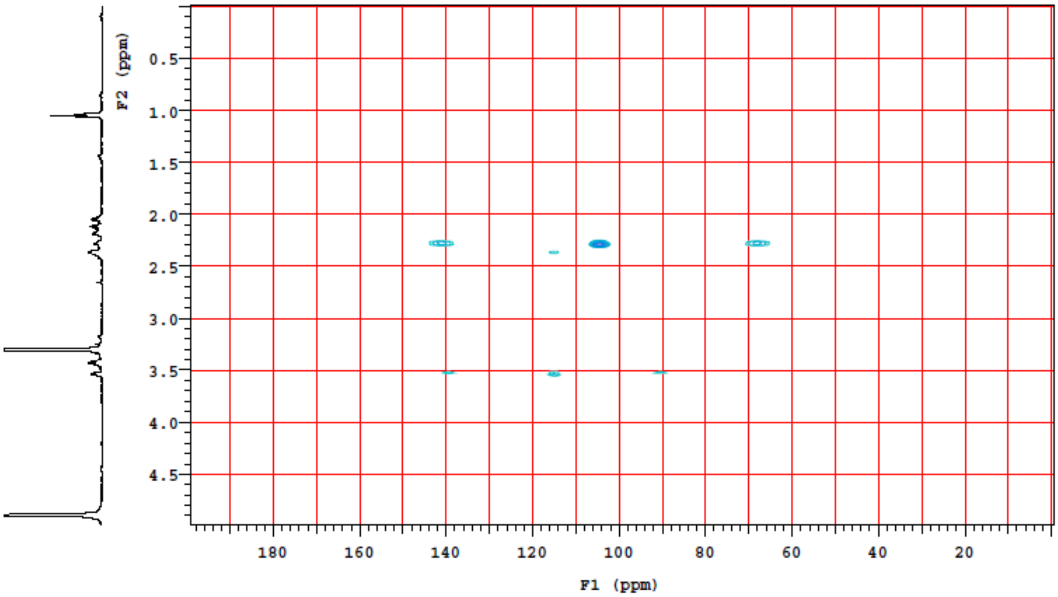

Supplement: Supplementary Information [file srep20340-s1.pdf]
